# Analysis of the factors influencing healthcare professionals’ adoption of mobile electronic medical record (EMR) using the unified theory of acceptance and use of technology (UTAUT) in a tertiary hospital

**DOI:** 10.1186/s12911-016-0249-8

**Published:** 2016-01-30

**Authors:** Seok Kim, Kee-Hyuck Lee, Hee Hwang, Sooyoung Yoo

**Affiliations:** Center for Medical Informatics, Seoul National University Bundang Hospital, Bundang Hospital, 166, Gumi-ro, Bundang-gu, Seongnam-si 436-707 South Korea

**Keywords:** Mobile EMR, Acceptance model, UTAUT, TAM, Structural equation model, Hospital information system

## Abstract

**Background:**

Although the factors that affect the end-user’s intention to use a new system and technology have been researched, the previous studies have been theoretical and do not verify the factors that affected the adoption of a new system. Thus, this study aimed to confirm the factors that influence users’ intentions to utilize a mobile electronic health records (EMR) system using both a questionnaire survey and a log file analysis that represented the real use of the system.

**Methods:**

After observing the operation of a mobile EMR system in a tertiary university hospital for seven months, we performed an offline survey regarding the user acceptance of the system based on the Unified Theory of Acceptance and Use of Technology (UTAUT) and the Technology Acceptance Model (TAM). We surveyed 942 healthcare professionals over two weeks and performed a structural equation modeling (SEM) analysis to identify the intention to use the system among the participants. Next, we compared the results of the SEM analysis with the results of the analyses of the actual log files for two years to identify further insights into the factors that affected the intention of use. For these analyses, we used SAS 9.0 and AMOS 21.

**Results:**

Of the 942 surveyed end-users, 48.3 % (23.2 % doctors and 68.3 % nurses) responded. After eliminating six subjects who completed the survey insincerely, we conducted the SEM analyses on the data from 449 subjects (65 doctors and 385 nurses). The newly suggested model satisfied the standards of model fitness, and the intention to use it was especially high due to the influences of Performance Expectancy on Attitude and Attitude. Based on the actual usage log analyses, both the doctors and nurses used the menus to view the inpatient lists, alerts, and patients’ clinical data with high frequency. Specifically, the doctors frequently retrieved laboratory results, and the nurses frequently retrieved nursing notes and used the menu to assume the responsibilities of nursing work.

**Conclusion:**

In this study, the end-users’ intentions to use the mobile EMR system were particularly influenced by Performance Expectancy and Attitude. In reality, the usage log revealed high-frequency use of the functions to improve the continuity of care and work efficiency. These results indicate the influence of the factor of performance expectancy on the intention to use the mobile EMR system. Consequently, we suggest that when determining the implementation of mobile EMR systems, the functions that are related to workflow with ability to increase performance should be considered first.

## Background

The worldwide expansion and growth of the mobile market has led to the formation of new markets in the majority of industries that are centered on mobile functionality [1–3]. The healthcare industry has exhibited a similar pattern, and services and applications that utilize mobile functionality are actively being developed in hospitals, organizations, and other groups [4, 5]. Examples of the growth of the mobile market include mobile electronic medical records (EMRs) that are used by healthcare professionals (medical staff), personal health record (PHR) applications that patients can use to examine and control their own health data, and applications that allow direct patient control over particular diseases. These applications reportedly improve the efficiency and effectiveness of hospitals and help to reduce organizational costs, which results in an essential element of hospital information systems (HISs) [6, 7].^6^, ^7^] However, when adopting these applications and mobile technologies, the functional features and advanced techniques have been focused on to a greater extent than the needs of and features for end-users [8, 9]. Consequently, low usage rates, resistance, abandonment of the use of health information technology (IT), and requests for alternative methods have occurred. Therefore, to elicit substantial effects, the reactions of end-users must be thoroughly considered [9].

The Technology Acceptance Model (TAM) is based on concepts from social psychology and is a tool for defining and testing the intentions of individual end-users to use new technology. In the industries other than healthcare, the TAM has been used as the gold standard [9]. Additionally, the end-user’s intention to use medical IT can be analyzed by utilizing the Unified Theory of Acceptance and Use of Technology (UTAUT) [10] model, which is a model that incorporates various models of human behavior theory. Studies that analyze end-user’s intentions to use medical IT are actively being conducted and include studies that utilize a single model, such as the TAM or the UTAUT, and studies that utilize combined hypotheses that suggest that the combination of many different existing models into an adjusted model is appropriate for a study [11–13]. However, the results that have been reported based on these analyses exhibit internal discrepancies that can be utilized to implement future studies following the confirmation of the characteristics of the applications and the end-users [9, 14].

In the present study, we analyzed the factors that influenced the intentions of healthcare professional (doctors and nurses) end-users to use a mobile EMR system that made patient medical information accessible on smart devices. Additionally, we confirmed the actual usage of the EMR by analyzing the log data that accumulated over two years at the observation site and compared this usage with the factors that influenced the intention to use.

### Related work

There have been various studies that have used the acceptance model to analyze end-users’ behavioral intentions to use a new technology in the healthcare field. However, discrepancies exist in the results of these studies that are based on the particular technologies and characteristics of end-users. Esmaeilzadeh P. et al. (2015) [15] analyzed the intention to use a clinical decision support system (CDSS) among 335 doctors in 12 hospitals and found that the influential factors were Performance Expectancy (PE), self-efficiency, and social network. Maillet É. et al. (2015) [16] studied the intention to use electronic patient records among 616 nurses from 4 hospitals and reported that PE was the most influential factor in terms of actual use and that Facilitating Conditions (FC) was the second highest one on Effort Expectancy (EE). Dunnebeil S. et al. (2012) [17] conducted research using a model that incorporated both the UTAUT and the TAM to confirm the degree of acceptance of e-health applications among German healthcare professionals. These authors found that both Perceived Usefulness (PU) and Perceived Ease of Use (PEOU) positively influenced the intention to use. Kijsanayotin B. et al. (2009) [18] used the UTAUT to analyze the degree of acceptance of medical IT in community health centers in Taiwan and found that PE, EE, Social Influence (SI) and Attitude (ATT) influenced the intention to use medical IT. Moreover, IT usage experience, FC, and the intention to use the system influenced the usage of medical IT in the Community Health Center (CHC). Heselmans A. et al. (2012) [19] used the UTAUT to confirm the factors that influenced the usage of EMRs among family physicians and reported that while PU, FC, and general satisfaction were correlated with the intention to use, PEOU and SI exhibited weak correlations with the intention to use. Other previous studies have analyzed users’ intentions to use new technologies, such as HIS [20], Picture Archiving and Communication System (PACS) [21], online information systems [22], remote medical programs [23], remote rehabilitation services [24], robotic operations by surgeons [25], and online health information [26].

### The problems and Our contributions

The abovementioned previous research has studied the intentions of end-users in terms of the successful adoption of new systems and technologies. However, these studies have suggested factors that influence the adoption of specific healthcare systems in terms of analyses that were based on specific theoretical models. Such results have not been verified in terms of the actual usages of new systems.

Therefore, this study aimed to contribute to the analysis of the theoretical factors that influence healthcare professionals’ adoption of mobile EMR systems using structural equation modeling (SEM) and to verify the results with log analyses of the 2-year usage patterns of a system in a real clinical environment.

### Healthcare IT: mobile EMR

Recently, many applications utilizing smartphones have been developed for a variety of healthcare fields, such as health, fitness, lifestyle, education, and management, and these applications are increasingly likely to include the advantages of on-board computing capabilities, large memory capacities, large screens and open operating systems [5]. Specifically, many hospitals have expressed great interest in mobile EMR systems. Because mobile EMR systems can be used anytime and anywhere, users are able to move quickly in response to communications to enhance helpfulness and the continuity of care [5]. However, mobile EMR systems need to consider factors related to the security of medical information due to their ability to function on personal smartphones [27].

### User acceptance models: UTAUT and TAM

**Fig. 1:**
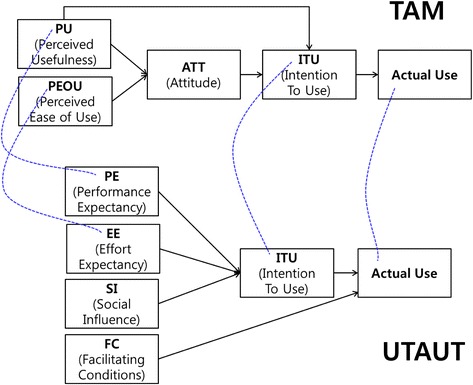
Comparison of the TAM and UTAUT models. The UTAUT was constructed by extracting 3 variables that influence behavioral intentions to use, 1 variable that influences action, and 4 control variables that mediate the effects of the process. Some of variables had similar concept with variables to construct the TAM

Whether end-users will actively accept and use a new technology when it is introduced has been a topic of research for many interested parties. The most widely used model for solving this research issue is the TAM that was suggested by Davis in 1989 [28]. This model has a basic framework in which the end-user’s attitudes regarding the use of new technology determine the user’s behavioral intention to use the technology at the actual site. However, because this model lacks a diversity of variables that influence the situation, the TAM has been modified to meet the necessities of each specific research project prior to its applications, or alternatively, only some of the variables of the TAM have been selected for analysis. To solve these problems, Venkatesh et al. (2003) [10] approached the end-user’s acceptance of technology from an integrated perspective and suggested the UTAUT, which has better explanatory power than the previous models (see Fig. 1).

The UTAUT [10] was constructed by extracting 3 variables that influence behavioral intentions to use, 1 variable that influences action, and 4 control variables that mediate the effects of the process. In other words, the UTAUT utilizes variables that influence the behavioral intention to use and suggests the use of PE, EE, and SI. FC has been suggested as a variable that directly influences action in addition to PE, EE, and SI, i.e., the variables that influence the behavioral intention to use. Additionally, the UTAUT suggests that these four independent variables influence the end-user’s behavioral intention to use. The 4 control variables, i.e., gender, age, experience, and voluntariness of use, exhibited mediating effects on the relationships between the influences of each variable.

The newly suggested UTAUT model is known to have 20 to 30 % greater explanatory power than the TAM, which on average exhibits 40 to 50 % explanatory power regarding the end-user’s behaviors or behavioral intention to use [10]. Therefore, the healthcare field is actively utilizing research on end-user acceptance that involves the hypothesis that was recently suggested by the UTAUT [9, 14–16, 29].

The remainder of the present paper is organized as follows: Section 2 contains our study design and methodology, Section 3 describes the results, Section 4 contains a discussion of our results and the limitations of the study, and Section 5 contains the conclusion.

## Methods

To identify and verify the factors affecting the adoption of the mobile EMR system, we confirmed the factors that influenced the end-users’ intentions to use the mobile EMR system via a theoretical analysis of our research model. After analyzing the usage logs over a timeframe of two years, we confirmed the usage status of the system and additionally compared the results with those from analyses of the factors that influenced the end-users’ intentions to use.

### Development of the mobile EMR system

This study was performed at a tertiary general university with a fully paperless comprehensive electronic health record (EHR) system in South Korea. This hospital has 1340 beds and an average of approximately 5000 outpatients daily as of August of 2013.

**Fig. 2:**
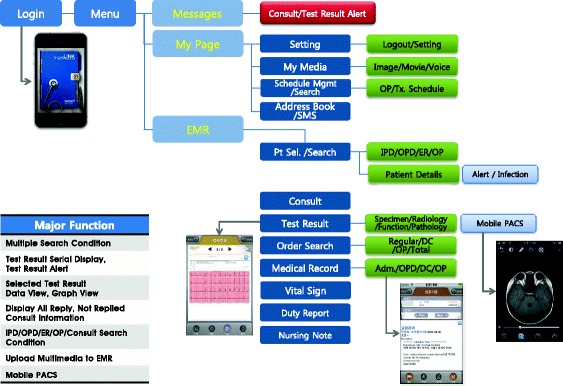
Function List and Screen Shots of the BESTCare LINK Mobile EMR Application. The BESTCare LINK system included functions that provided notifications of consultation requests and the completions of examination results to the users and allowed of the selection of patients to check orders, examination results, medical records, and PACS images

The hospital launched a mobile EMR system named BESTCare LINK on March 26, 2012 following the implementation of this system for approximately 9 months beginning in June of 2011. The BESTCare LINK included most of the functions for viewing EHR data (e.g., medical records, PACS images, and examination results) in mobile settings (see Fig. 2). However, this system did not allow for the writing of orders or records to protect personal information.

The BESTCare LINK system was developed to consider both the functional aspects required by the end-user and the security of the system. All smartphone, tablet, and PC devices that use iOS or Android OS were able to access the BESTCare LINK system.

### Hypothesis

**Fig. 3:**
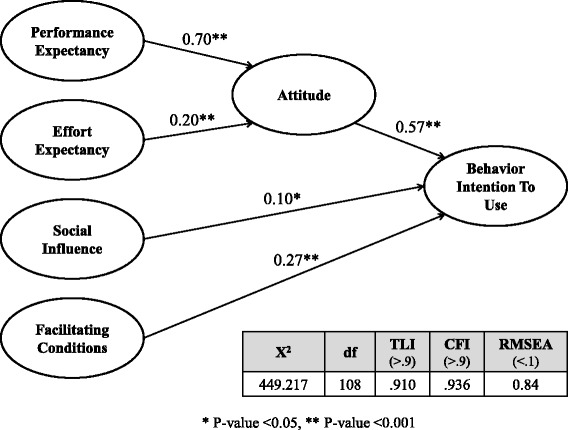
Results of the End-User Acceptance Model for the Mobile EMR System. The model significantly satisfied the standards. The relationships between the latent variables exerted positive influences that aligned with the research hypotheses, and these influences were statistically significant. Specifically, the influences of PE on ATT (0.49=0.70^2^) and ATT on the Behavior Intention to Use (0.325=0.57^2^) were stronger than the other relationships between the latent variables

Recently, mobile devices and applications have been selected for use based on the user’s characteristics and preferences [30]. In other words, even when a new device featuring new technology comes out, end-users purchase and use a device only when they decide based on individual preference that the product is good. Therefore, to effectively analyze the end-user’s intention to use mobile devices, the end-user attitude must be thoroughly considered. In the present study, we used the UTAUT model as the basic framework to analyze the end-users’ intention to use mobile EMR [31], and we used a model that integrated the concepts of the ATT of the TAM in the final analysis to analyze the end-users’ ATTs while considering the characteristics of mobile application (see Fig. 3).

The UTAUT suggests that PE, EE, and SI positively influence the behavioral intention to use and that FC positively influences the actual action [10]. However, due to the difficulty of investigating the actual actions, many studies have hypothesized that FC has an influence on behavioral intention to use that is similar to those of other independent variables [19, 21, 32, 33]. Similarly, this study was not able to investigate the actual actions and thus hypothesized that FC exerted a positive impact on behavioral intention to use. Moreover, we applied the concepts that PEOU and PU positively influence ATT to this research model as suggested in the TAM, [28] and we hypothesized that PE and EE also exerted positive influences on ATT because PE and EE in the UTAUT denote the same concepts as PEOU and PU in the TAM. Finally, we applied the concept of ATT that is suggested in the TAM model, i.e., ATT has a positive influence on the behavioral intention to use, and derived the following hypotheses about the model for analysis in the present study. The actual use variable could not be examined, even though we have log files that represent actual use, because the questionnaire survey of the end-users’ intentions to use did not include questions regarding the actual use variable. Furthermore, the survey was conducted anonymously. Therefore, we could not examine the survey respondents’ actual usage log.

- H1: PE in the Mobile EMR will positively influence ATT.
- H2: EE in the Mobile EMR will positively influence ATT.
- H3: ATT in the Mobile EMR will positively influence the intention to use.
- H4: SI in the Mobile EMR will positively influence the intention to use.
- H5: FC in the Mobile EMR will positively influence the intention to use.

### Questionnaire survey

We conducted a survey of the end-users’ intentions to use the BESTCare LINK system between the approximately two weeks from October 12 to 26 in 2012 with doctors and nurses who had experience using the BESTCare LINK system. We conducted the survey by distributing paper-based questionnaires that were structured in a self-report format to the appropriate departments and collected the questionnaires from those departments. We conducted the survey anonymously and asked no personal identifiers that allow identification of the responder. Thus, the use of the survey response data was approved by the Seoul National University Bundang Hospital’s institutional review board.

For the survey tools, we integrated the UTAUT model and a part of the TAM model and subsequently created questionnaires to analyze the factors that influenced adoption. After the first round of translations, healthcare professionals (i.e., doctors and nurses), healthcare information experts, and researchers performed an additional round of review. Next, we extracted 17 questions from the UTAUT questionnaire (3 PE questions, 3 EE questions, 2 ATT questions, 3 SI questions, 3 FC questions, and 3 questions regarding the intention to use). To this set of questions, we added 1 question regarding the overall satisfaction with the mobile EMR system and 10 questions regarding the expected effectiveness of the system. Thus, the final questionnaire comprised 28 questions. We used SAS 9.2 for the statistical analyses of the questionnaire, which included a five-point response scale, and we used AMOS 21 for the SEM analyses. To verify the measurements of the concepts that comprised the SEM, we used convergent validity and discriminant validity, and we used maximum likelihood estimation (MLE).

### Analysis of the mobile EMR system Log

We analyzed the usage log for the mobile EMR system, which contained two years of data from May of 2012 to April of 2014. The first month (April of 2012) was hypothesized to be an application adaptation period and was eliminated from the analyses. We investigated the log to discover trends regarding the actual usage of the BESTCare LINK system, the overall usage patterns, and the usage characteristics of certain user groups. We also carefully observed whether there were user groups who used the mobile EMR system more effectively than other groups. The event log of the mobile EMR system was automatically stored in a database with the information of the user, the event time, and the access page. The data were extracted into Microsoft Access 2010, and the frequency analyses were conducted with SAS 9.2.

## Results

### Demographics of the questionnaire respondents

The survey was conducted over two weeks, and a total of 455 subjects, including 97 doctors (23.2 % response rate) and 358 nurses (68.3 % response rate), responded. Six subjects were eliminated from the analyses due to incomplete surveys. Therefore, we conducted the analyses based on the surveys of 449 subjects. The subjects comprised 65 men (14.5 %) and 384 women (85.5 %) and included 94 doctors (20.9 %) and 355 nurses (79.1 %). Thus, the proportions of women and nurses were high (Table 1).

**Table 1:** Demographics of the questionnaire respondents

Demographic		N(%)
Occupation	Doctor	94(18.8)
	Nurse	355(71.1)
Gender	Male	65(13.0)
	Female	384(76.9)
Working Years	≤ 3 year	222(44.5)
	≤ 7 year	125(25.1)
	≤ 10 year	95(19.0)
Age Group	20 s	228(45.6)
	30 s	181(36.2)
	40 s	32(6.4)
	50 s	7(1.4)
	≥ 60 s	1(0.2)
Mobile IT familiarity	Very unfamiliar	15(3.0)
	Unfamiliar	45(9.0)
	Neutral	186(37.2)
	Familiar	155(31.0)
	Very familiar	47(9.4)
Voluntary use	Strongly disagree	46(9.2)
	Disagree	80(16.0)
	Neutral	125(25.0)
	Agree	142(28.4)
	Strongly agree	56(11.2)

### Evaluation of the results from the New model of mobile EMR use based on SEM analysis

Of the 28 survey questions, the UTAUT questionnaire was composed of 17 questions and 6 constructs. To confirm the consistency of the items that were being measured prior to verifying the model and the hypothesis of the SEM analysis, we calculated the Cronbach’s α. This analysis of reliability revealed that the Cronbach’s αs for all of the variables exceeded 0.8. Thus, there were no issues related to the reliabilities of the measured variables (Table 2).

**Table 2:** Reliability analysis

Construct	Item’s no.	Cronbach’s α (>0.7)
Performance expectancy	3	0.896
Effort expectancy	3	0.928
Attitude	2	0.872
Social influence	3	0.844
Facilitating conditions	3	0.808
Behavior intention to use	3	0.979

To analyze the SEM, we conducted a process that first involved the confirmation of the reliabilities and validities of the constructs, including the constructs’ convergent and discriminant validities. Convergent validity is an evaluation on the consistency of the observed variables that measure the latent variables. To analyze the convergent validity, we confirmed values, including the standardized factor loading, significance, average variance extracted (AVE), and construct reliability (CR). As shown in Table 2, the loadings and weights of all of the constructs (i.e., PE, EE, ATT, SI, FC, and Behavior Intention to Use), as well as the t-value, CRs, and AVE values were analyzed to determine whether they satisfied the standards, and we decided that there were no issues with the convergent validities of the constructs (Table 3).

**Table 3:** Convergent validity

Construct	Item	Load/Weight (>0.7)	t-value (>1.965)	CR (>0.7)	AVE (>0.5)
Performance expectancy	PE1	0.805	22.020	0.929	0.813
	PE2	0.902	26.891		
	PE3	0.893	fix		
Effort expectancy	EE1	0.860	26.176	0.949	0.860
	EE2	0.959	32.309		
	EE3	0.894	fix		
Attitude	ATT1	0.858	24.196	0.899	0.817
	ATT2	0.901	fix		
Social influence	ATT1	0.884	16.160	0.896	0.743
	SI1	0.852	15.904		
	SI2	0.694	fix		
Facilitating conditions	F1	0.694	13.499	0.830	0.623
	F2	0.903	15.941		
	F3	0.707	fix		
Behavior intention to use	BI1	0.960	fix	0.983	0.951
	BI2	0.973	55.327		
	BI3	0.977	56.612		

^a^CR: composite reliability, AVE: average variance extracted

The discriminant validity indicates the extent to which the latent variables are distinct from one another. Some of the methods for calculating discriminant validity include comparing the AVE values and squared correlation coefficients of two constructs (AVE > correlation coefficient ^2^) and verifying that the result of calculating the correlation coefficient between two constructs ± 2 multiplied by the standard error (SE) does not include 1 (1 ≠ correlation coefficient ± 2 × SE). When analyzing the convergent validity, if the values satisfy the standard values, and the values of the correlation coefficients are greater than 0.4 and less than 0.6, it is then possible to analyze the discriminant validity results that satisfy the criteria of both methods without performing additional calculations. However, although most of the correlation coefficients between constructs were within the range of 0.4 to 0.6, the relationship between ATT and PE and the relationship between Behavioral Intention To Use and ATT exhibited relatively high correlation coefficients (Table 4). When we used the two aforementioned methods to analyze the discriminant validity, the results appeared to be appropriate in terms of the standards. In other words, our analysis determined that the discriminant validity did not exist and that the relevance of the relationships between the constructs were adequate.

**Table 4:** Discriminant validity

	Performance expectancy	Effort expectancy	Attitude	Social influence	Facilitating conditions	Behavioral intention to use
Performance expectancy	1					
Effort expectancy	0.550	1				
Attitude	0.722^a^	0.571	1			
Social influence	0.569	0.446	0.563	1		
Facilitating conditions	0.398	0.547	0.509	0.404	1	
Behavior intention to use	0.610	0.546	0.746^a^	0.564	0.589	1
Cronbach’s α	0.896	0.928	0.872	0.844	0.808	0.979
CR	0.929	.0949	.0899	0.896	0.830	0.983
AVE	0.813	0.860	0.817	0.743	0.623	0.951

^a^Correlation: equal to or more than 0.7

† All correlation coefficients between factors were significant at *P*-value < .01

### Factors influencing healthcare professionals’ adoption of the mobile EMR

In this study, we analyzed the influences of PE, EE, SI, and FC on the Behavioral Intention To Use and the mediating effects of ATT. Fig. 3 illustrates the research model that was followed for the SEM analysis. The fit indices of the research model were calculated as *X* ^2^ =449.217 (df=108, *p*=0.000), TLI=0.910, CFI=0.936, and RMSEA=0.84. Because the model satisfied the standards of the TLI and CFI exceeding 0.9 and the RMSEA being below 0.1, and the model’s *P*-value was statistically significant, this model was found to be adequate for analyzing the end-user’s intention to use the mobile EMR system. The relationships between the latent variables had a positive influence, which aligned with the research hypotheses, and this influence was statistically significant. Specifically, influences of PE on ATT (0.49=0.70^2^) and ATT on Behavioral Intention to Use (0.325=0.57^2^) were found to be stronger than the other relationships between the latent variables.

### Questionnaire analysis results regarding the expected effectiveness of the mobile EMR system

Additionally, the overall satisfaction and expected effectiveness of the use of the BESTCare LINK system were compared according to occupation. The overall average score was greater than 3 (out of 5), and the doctors gave slightly higher scores than the nurses. Specifically, both the doctors and nurses reported the highest possible satisfaction score for the following two items: “To easily check the patient’s condition and test results at any time,” and “Useful for managing inpatients.” Examinations of the discrepancies in satisfaction scores according to occupation revealed that the doctors reported higher satisfaction scores than the nurses for all of the questions, but we observed a significant difference for the expected effectiveness question, “To help in quick decision making” (Table 5). We concluded that overall, the subjects were satisfied with the functions of the BESTCare LINK system without significant differences by occupation. However, regarding decision making, the doctors were slightly more satisfied with the BESTCare LINK system than the nurses.

**Table 5:** Expected effectiveness of the use of the mobile EMR system

Expected effectiveness questionnaire	Doctor		Nurse		*P*-value	Total
Overall satisfaction of mobile EMR system	3.7	(1.067)	3.6	(0.891)	0.732	3.6(0.931)
To check easily the patient’s condition and test results at anytime	3.9	(0.897)	3.8	(0.889)	0.542	3.8(0.891)
Useful for managing inpatients	3.9	(0.870)	3.8	(0.838)	0.363	3.9(0.845)
To determine the treatment and respond quickly	3.8	(0.934)	3.7	(0.893)	0.268	3.7(0.903)
Useful for communication among clinical staffs	3.6	(0.891)	3.4	(0.930)	0.074	3.5(0.925)
To help in quick decision-making	3.8	(0.882)	3.4	(0.909)	0.001*	3.5(0.915)
Positive impact on my working patterns	3.8	(0.904)	3.6	(0.888)	0.172	3.7(0.893)
Helpful to explain to patients	3.4	(0.868)	3.3	(0.907)	0.294	3.3(0.900)
(Doctor) To respond quickly through checking the referral quickly	3.5	(1.019)	-		-	3.5(1.019)
(Doctor) Useful for checking the outpatient in advance	3.3	(1.006)	-		-	3.3(1.006)
(Nurse) Useful for the change-over	-		3.5	(0.913)	-	3.5(0.913)

*P-value < 0.01

### Results of the analysis of the mobile EMR system Log

**Fig. 4:**
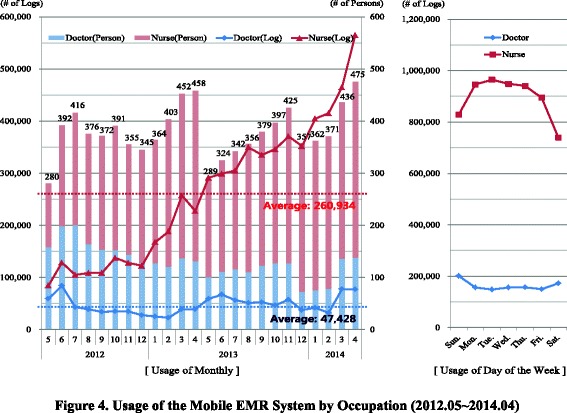
Usage of the Mobile EMR System by Occupation (2012.05–2014.04). After analyzing the user log data of BESTCare LINK system that were collected over two years, we found that the usage of doctors fluctuated little, whereas the usage of nurses consistently increased (Fig. 4). When the average usages on weekdays and weekends were compared, the weekend usage by doctors was found to be 21.7 % higher, and the weekend usage by nurses was found to be 16.5 % lower

After analyzing the user log of the BESTCare LINK system that was collected over two years, we found that the usage by doctors was consistent and exhibited little fluctuation, while the usage by the nurses consistently increased (Fig. 4). In terms of usage, the numbers of doctor and nurse users calculated monthly exhibited similar patterns. In May of 2013, the number of end-users decreased significantly for a brief period because the study site launched a new EMH system. However, the rate of total log counts did not decrease, which was suggestive of a high degree of loyalty among the main users of the mobile EMR system. When we examined the monthly usage of the system according to occupation, we found that 260,000 log-ins were by nurses, and 47,000 log-ins were by doctors. The average numbers of end-users per month were 249 for the nurses and 130 for the doctors. Moreover, the average numbers of uses per month were 1047 for the nurses and 363 for the doctors. Therefore, we presume that the EMR system helped the actual work of the nurses, which indicates that nurses should be considered major target users of mobile EMR systems.

When the entire two years of usage was compared in terms of the number of log-ins per day, discrepancies between the occupations were observed. The doctors exhibited lower usage frequencies on weekdays (i.e., Monday through Friday) compared with the weekends (i.e., Saturday and Sunday). The nurses exhibited higher usage frequencies on weekdays compared with weekends. When the average usages on the weekdays and the weekends were compared, the weekend usage by doctors was 21.7 % higher, and the weekend usage by nurses was 16.5 % lower. Thus, there seemed to be differences in the usage patterns of the doctors and nurses.

**Fig. 5:**
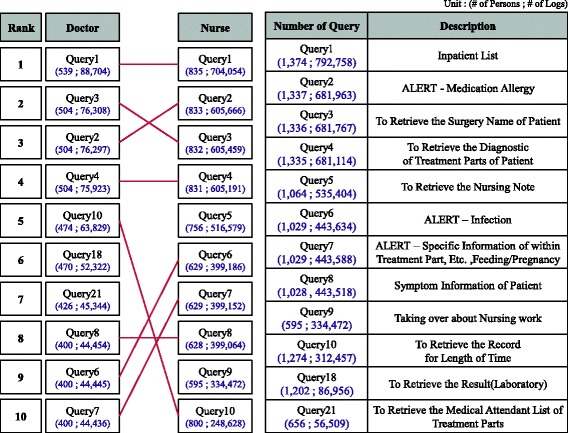
List of the 10 Most Frequent Queries of the Mobile EMR System Over 2 years. A list of the highest frequency queries (data requests) of the BESTCare LINK system, and lists of the 10 highest frequency queries according to occupation. The most frequent queries of the doctors were related to care, and queries that differentiated the doctors from the nurses were related to care and actual work processes, for example, queries related to viewing test results and the assignments of doctors by department

Fig. 5 provides a list of high-frequency queries (data requests) that occurred during the use of the BESTCare LINK system and lists of the top 10 queries by occupation. The queries of the doctors and nurses primarily exhibited similar patterns. However, the majority of the queries were categorized within the top 10 most frequent queries of both the doctors and the nurses, although some of the queries occurred only in the respective occupational duties. Consequently, requests for lists of the hospitalized patients (query no. 1) comprised the most widely used query and were accessed by 539 doctors (88,704 times) and 835 nurses (704,054 times). Some queries occurred with similar frequencies, such as medication allergy alert queries (query no. 2), patient surgery-related queries (query no. 3) and diagnosis confirmation queries (query no. 4). However, nurse note views (query no. 5) and nurse handover queries (query no. 9) were functions that were used exclusively by the nurses and seemed to occur frequently because these queries supported the effective completion of regular work processes. Regarding the doctors, the majority of the queries were related to care, and the queries that differentiated the doctors from the nurses were queries related to care and actual work procedures, such as queries to view test results and the assignments of doctors by departments.

## Discussion

This study aimed to identify and verify the factors related to the adoption of a mobile EMR system by healthcare professionals by using the SEM analysis of questionnaire survey data and an analysis of system log data that reflected the actual usage by the end-users.

First, we adjusted a model to be appropriate for analyzing end-users’ intentions to use a mobile EMR system by incorporating the concepts of the TAM and UTAUT. Based on the SEM analysis method, our new model revealed a greater influence of PE on ATT and a greater influence of ATT on the behavioral intention to use, and these results indicated that a stronger relationship between a new technology and work performance was associated with a greater influence on the end-users’ behavioral intention to use. Thus, it seems that the end-users intended to use the mobile EMR system to improve their work efficiency.

Second, the results of the analyses of the actual log of activity over two years revealed that the usage by doctors fluctuated little and that the usage by nurses exhibited a constant increase over the two years. Moreover, both the doctors and nurses frequently used the ‘Inpatient List’ and ‘Alert’ menus. Regarding the retrieval of patient data, the doctors frequently retrieved patients’ laboratory results, and the nurses frequently retrieved nursing notes and used the menu to take over their nursing work. These functions were important for the doctors and nurses to check patients’ statuses and make clinical decisions. Specifically, the nurses were able to check the statuses of their patients in advance of beginning work; thus, their take-over times could be reduced by using the mobile EMR system. It seems that these functions satisfied the needs of the end-users and enhanced their work performances.

The results of the analyses of the actual logs were similar to those of the SEM analyses in that PE was the most influential factor. Similar to our study, other studies have suggested new models based on the integration of various theories [4, 17, 30], and other studies have found that PE affects the intention to use [4, 17, 18, 21, 34]. In contrast, one study [35] used a single model, and another study [19] found that PE did not affect the intention to use. Although those previous studies were performed to identify the factors that influence the intention to use, we sought to verify the effect of the relationship of the end-users’ usage features with the influential PE factor on the intention to use the Mobile EMR system with both SEM and system log analyses.

Based on our study, it seems that mobile EMR systems are directly related to improvements in the performances of healthcare professionals. Thus, performance improvements affect the intentions of healthcare professionals to use mobile EMR systems because such healthcare systems have been used to improve the quality of care and work efficiency. Therefore, healthcare organizations should consider PE as a determining factor in the adoption of new mobile EMR systems and should deeply analyze the end-users’ needs to identify useful functions for their workflows.

The limitations of this study include the fact that the actual use variable was excluded from our analysis and that this study was performed in a single site and thus did not consider the characteristic differences of various medical and healthcare organizations. Moreover, all of the factors, such as PE, EE, SI, FC, and ATT, could not be compared and explained with the system log data because the log data included only the end-users’ system usages.

Future work needs to departmentalize the factors that influence the intention to use mobile EMR systems and to make various hypotheses regarding the relationships between those factors to identify additional influential factors. Additionally, the analyses of long-term log data will be helpful for generalizing the end-users’ characteristics and usage patterns, identifying the end-users’ actual needs, and elucidating the relationship between the intention to use and actual use. Additionally, it is necessary to conduct further studies regarding the possibility of generalizing mobile EMR systems in the future.

## Conclusion

This study suggested a new model for the intentions of healthcare professionals to use a mobile EMR system and revealed that the end-users exhibit positive intentions to use and positive attitudes toward a system if it helps their work performance.

In the future, this model needs to be verified with more varied end-users and hospitals, and the relationships of clinical factors with the factors that influence the behavioral intention to use a mobile EMR system require further investigation.

## Abbreviations

ATT: Attitude
AVE: Average Variance Extracted
CDSS: Clinical Decision Support System
CHC: Community Health Center
CR: Construct Reliability
EE: Effort Expectancy
EHR: Electronic Health Record
EMR: Electronic Medical Record
FC: Facilitating Conditions
HIS: Hospital Information System
IT: Information Technology
MLE: Maximum Likelihood Estimation
PACS: Picture Archiving and Communication System
PE: Performance Expectancy
PEOU: Perceived Ease of Use
PHR: Personal Health Record
PU: Perceived Usefulness
SE: Standard Error
SEM: Structural Equation Modeling
SI: Social Influence
TAM: Technology Acceptance Model
UTAUT: Unified Theory of Acceptance and Use of Technology
